# Supplementary figures and images for: FMRP and MOV10 regulate Dicer1 expression and dendrite development
Source: PLoS One. 2021 Nov 30;16(11):e0260005. doi: 10.1371/journal.pone.0260005 (PMC8631628; doi:10.1371/journal.pone.0260005)

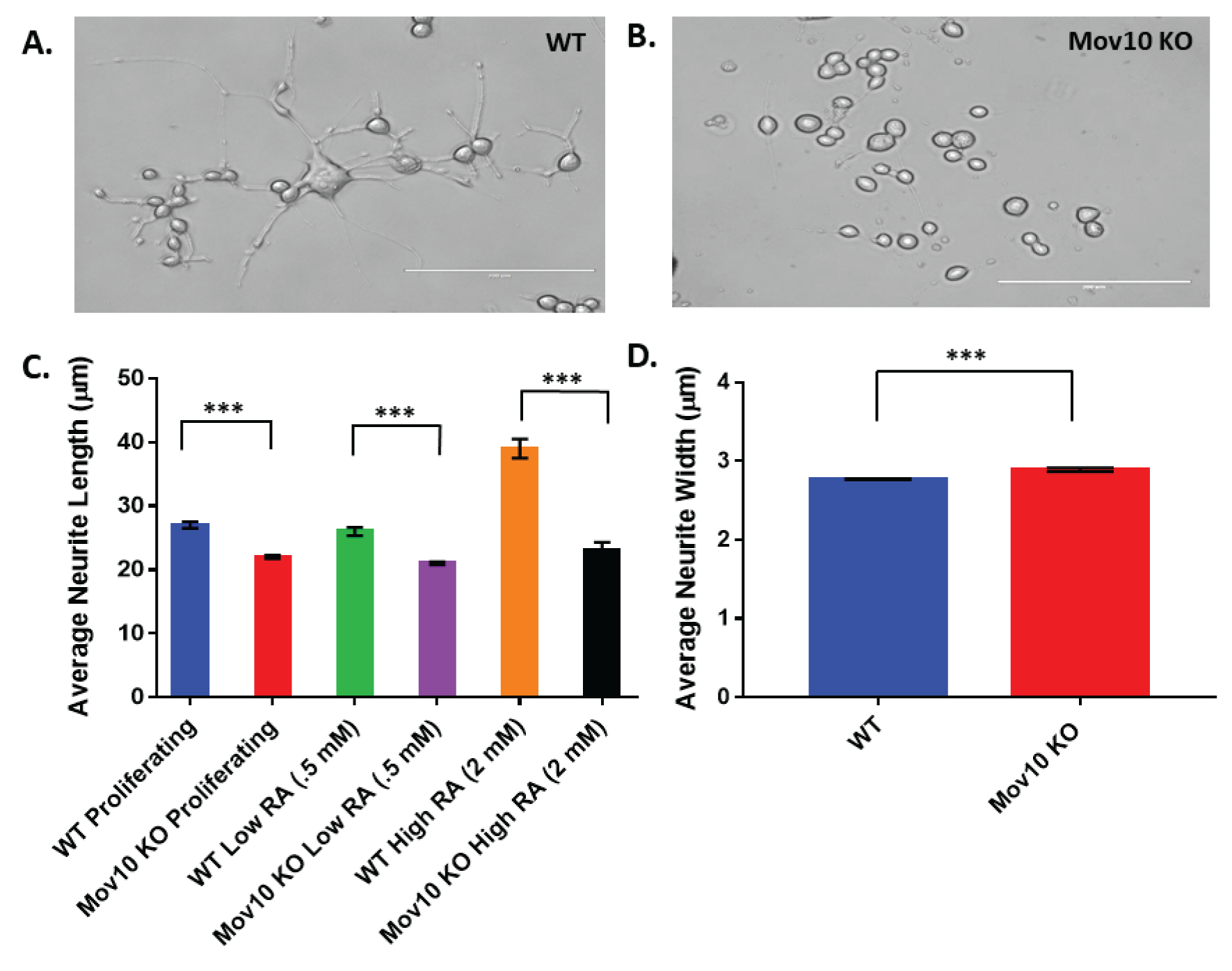

Supplement: S1 Fig — (A, B). Brightfield images of N2A WT and Mov10 KO cells. Scale bar = 200 μm. (C). Quantification of neurite length of WT and Mov10 KO in the presence of different concentrations of retinoic acid (RA). Between 800–1000 proliferating and differentiated cells were counted from triplicate experiments, and a total of 10 images were counted per condition. (D). Average neurite width in differentiated WT and Mov10 KO N2A cells (1 mM RA) were measured n = 100–250 (WT and Mov10 KO). All measured data are expressed as means ± SEM. ***p < 0.001 (Student’s t-test with Welch’s correction). (TIF) [file pone.0260005.s001.tif]

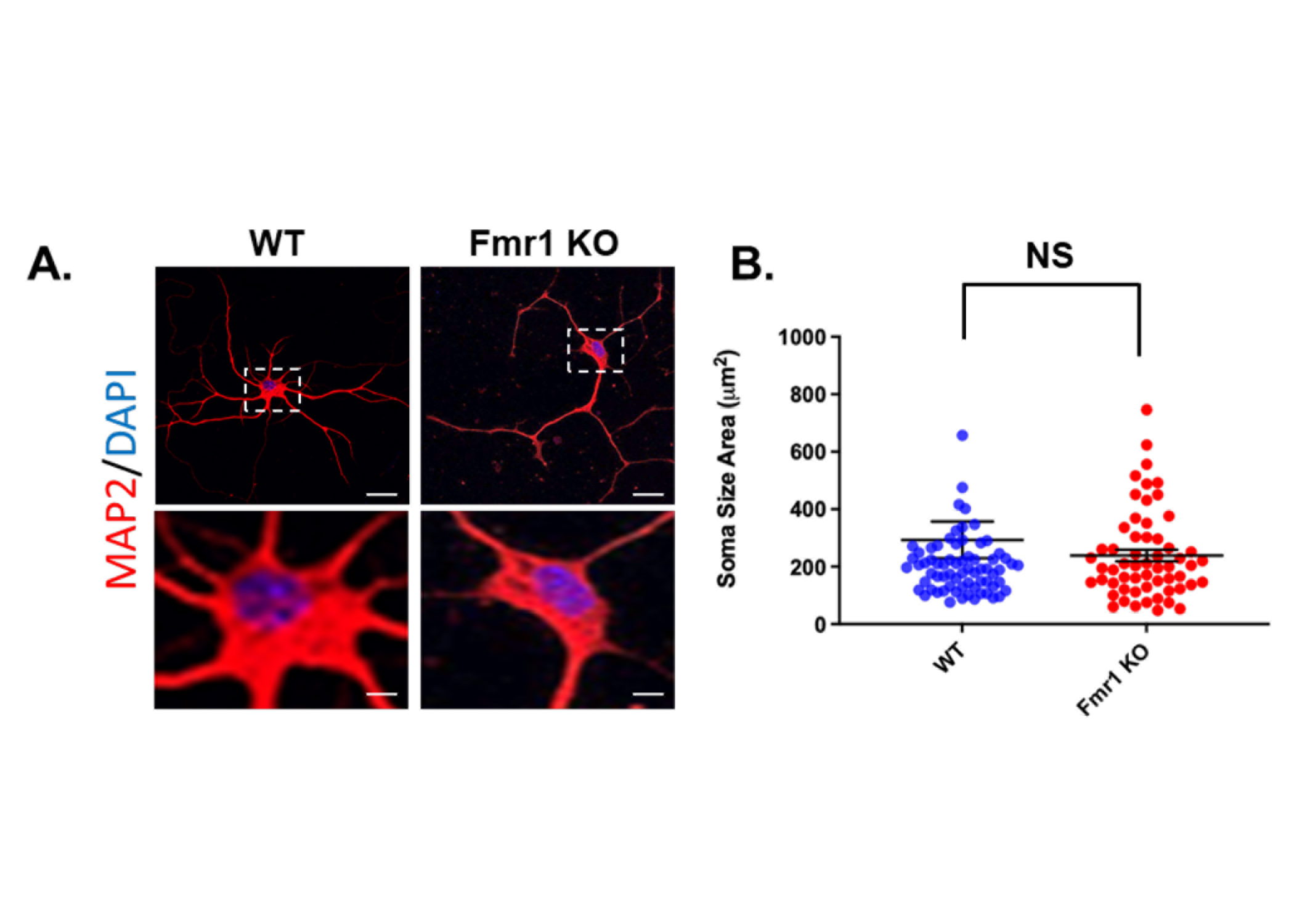

Supplement: S2 Fig — (A) Immunofluorescence microscopy of control (WT) and Fmr1 KO primary hippocampal neural cultures at 14 days in vitro (DIV14) showing MAP2 (red) and DAPI (Jentarra et al., 2010). The dashed box indicates the region shown at higher magnification. (B) Measurements of soma size area in DIV14 primary hippocampal neurons in Fmr1 KO (n = 58) compared to WT (n = 56). Scale bar: 25 μm. Data are presented as mean ± SEM; p values in relation to control (NS = p > 0.05), (Student’s t-test with Welch’s correction). (TIF) [file pone.0260005.s002.tif]

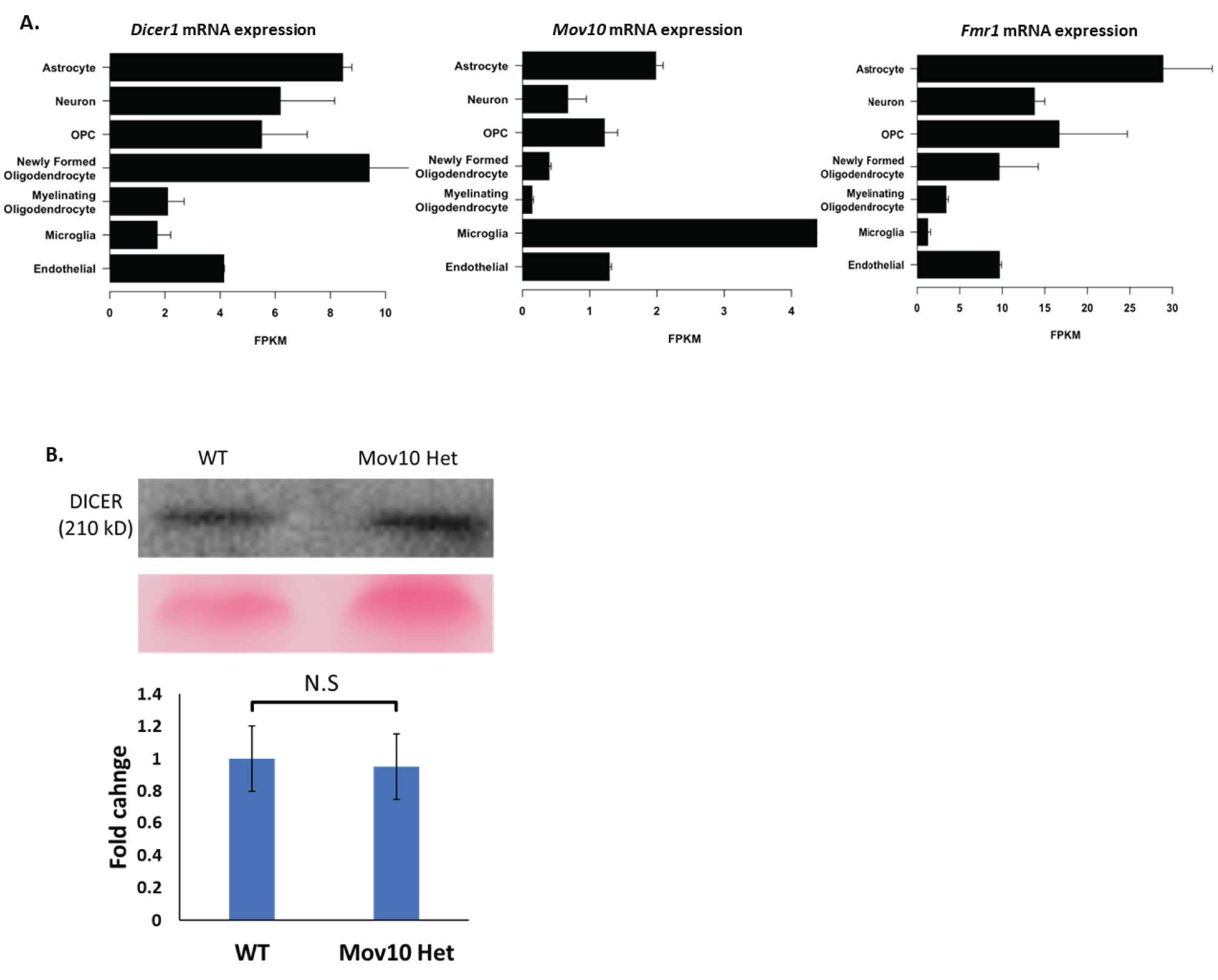

Supplement: S3 Fig — A) Whole P2 WT and Mov10 Het (25 μg) were immunoblotted against DICER with Ponceau S as a loading control in three independent experiments. Error bars represent SD, and p values were obtained by Student’s t test with Welch’s correction (NS > 0.05). (TIF) [file pone.0260005.s003.tif]

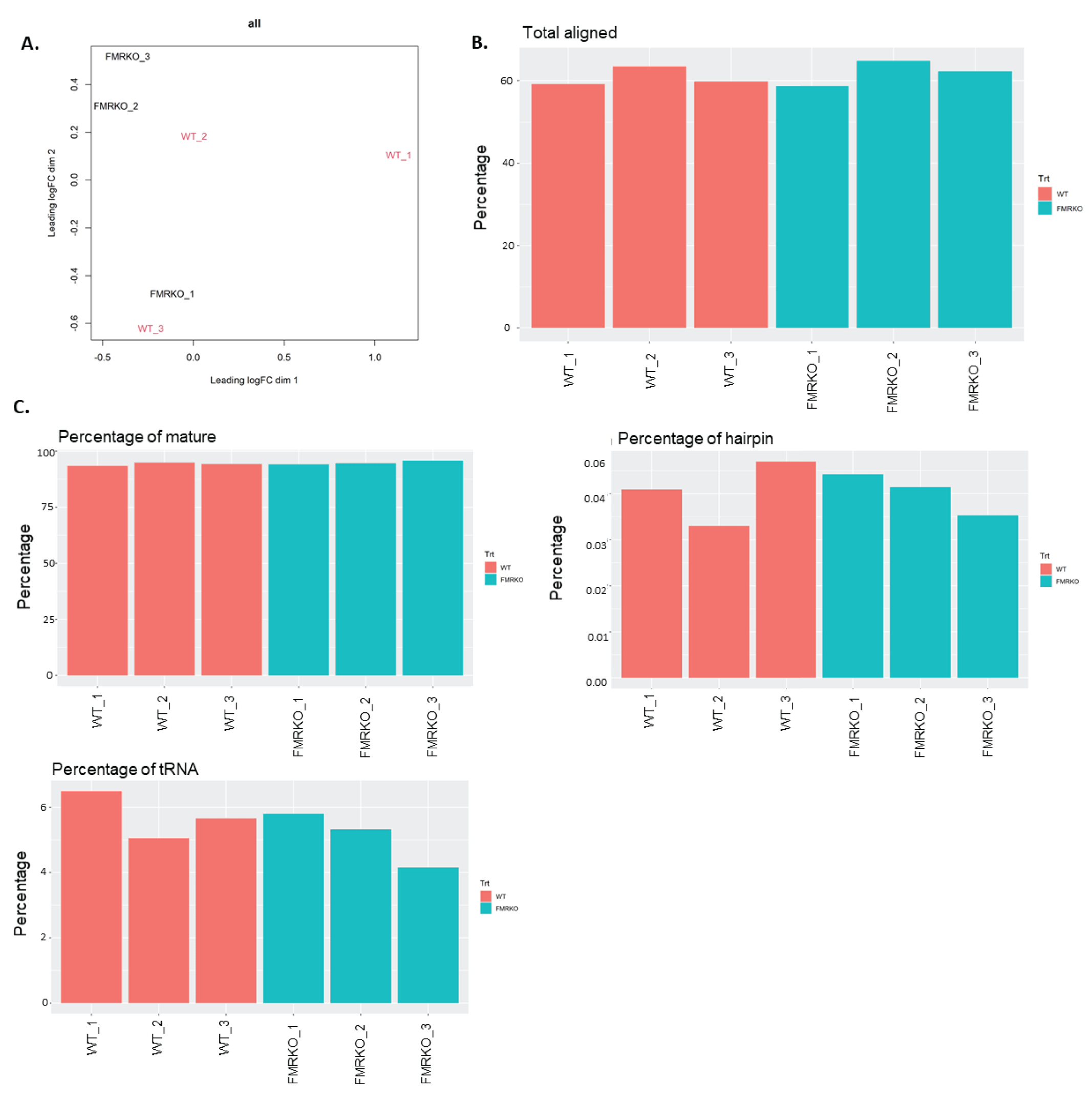

Supplement: S4 Fig — A). Multidimensional scaling on the top 500 most variable genes in brain samples. B). Percentages of total reads in the sequencing report that mapped to any of these three: mature, hairpin and tRNA ranged from 58.6 to 64.8%. The mapping percentage does not differ between WT and FMRKO samples. C). Percentage of mapped reads coming from mature miRNA, hairpin miRNA, and tRNA. (TIF) [file pone.0260005.s004.tif]

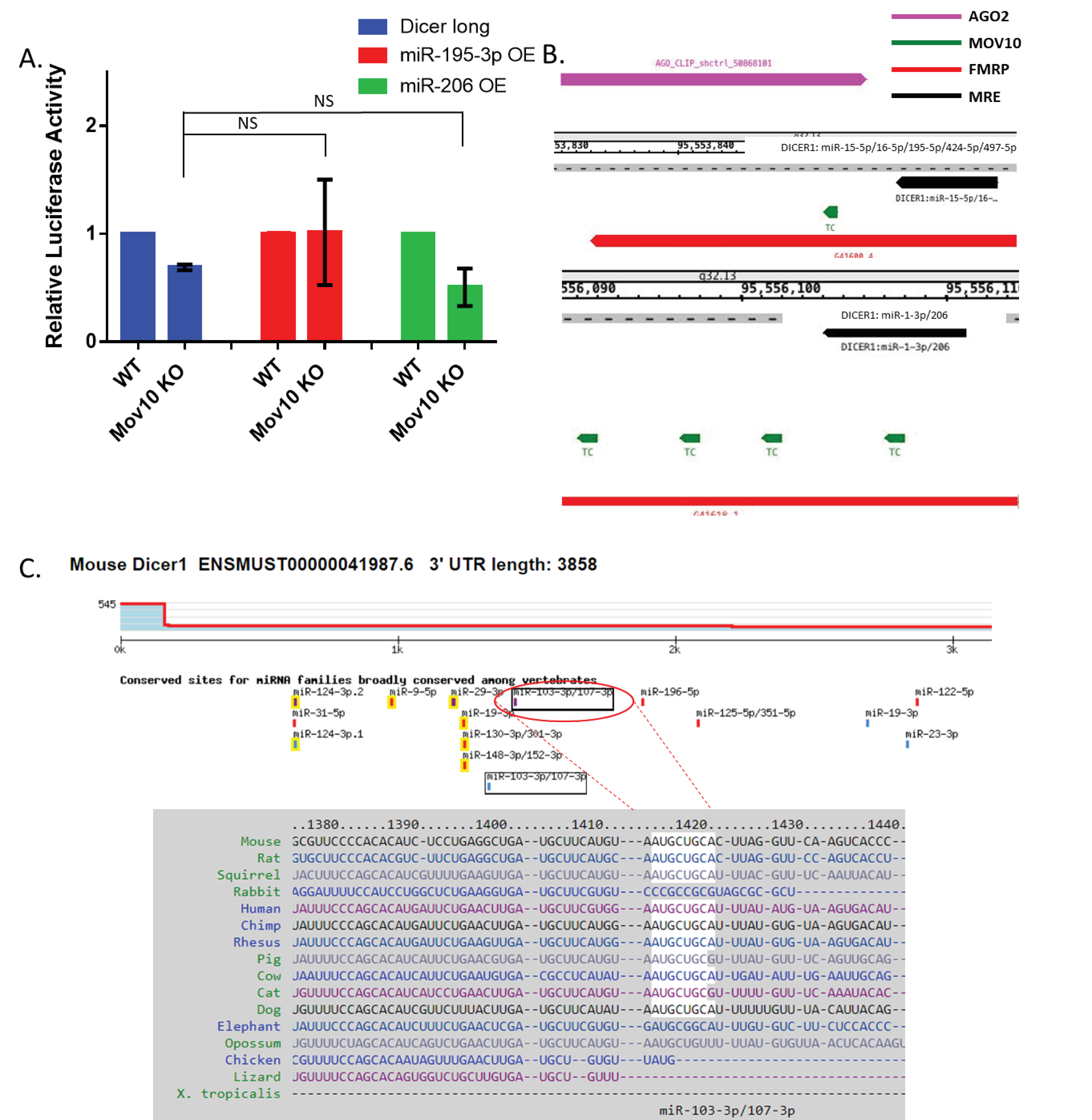

Supplement: S5 Fig — (A). Effect of Mov10 KO on luciferase Dicer1 3′ UTR and miR-195-3p and miR-206 site overexpression. (B) Screenshot from Integrated Genome Browser (IGB) of Dicer1 3’UTR (running from right to left) with human AGO2 CLIP sites (pink), human MOV10 CLIP sites (green) and human FMRP CLIP sites showing relative locations to the MRES containing sites for miR-195-3p and miR-206 binding. (C). TargetScan screenshot of Dicer1 3’UTR (running from right to left) with red circles showing location of MRE sites whose miRNAs were overexpressed in A. Assays were performed in quadruplicate from three independent experiments. All measured data are expressed as means ± SEM. ***p < 0.001 (Student’s t-test with Welch’s correction). (TIF) [file pone.0260005.s005.tif]

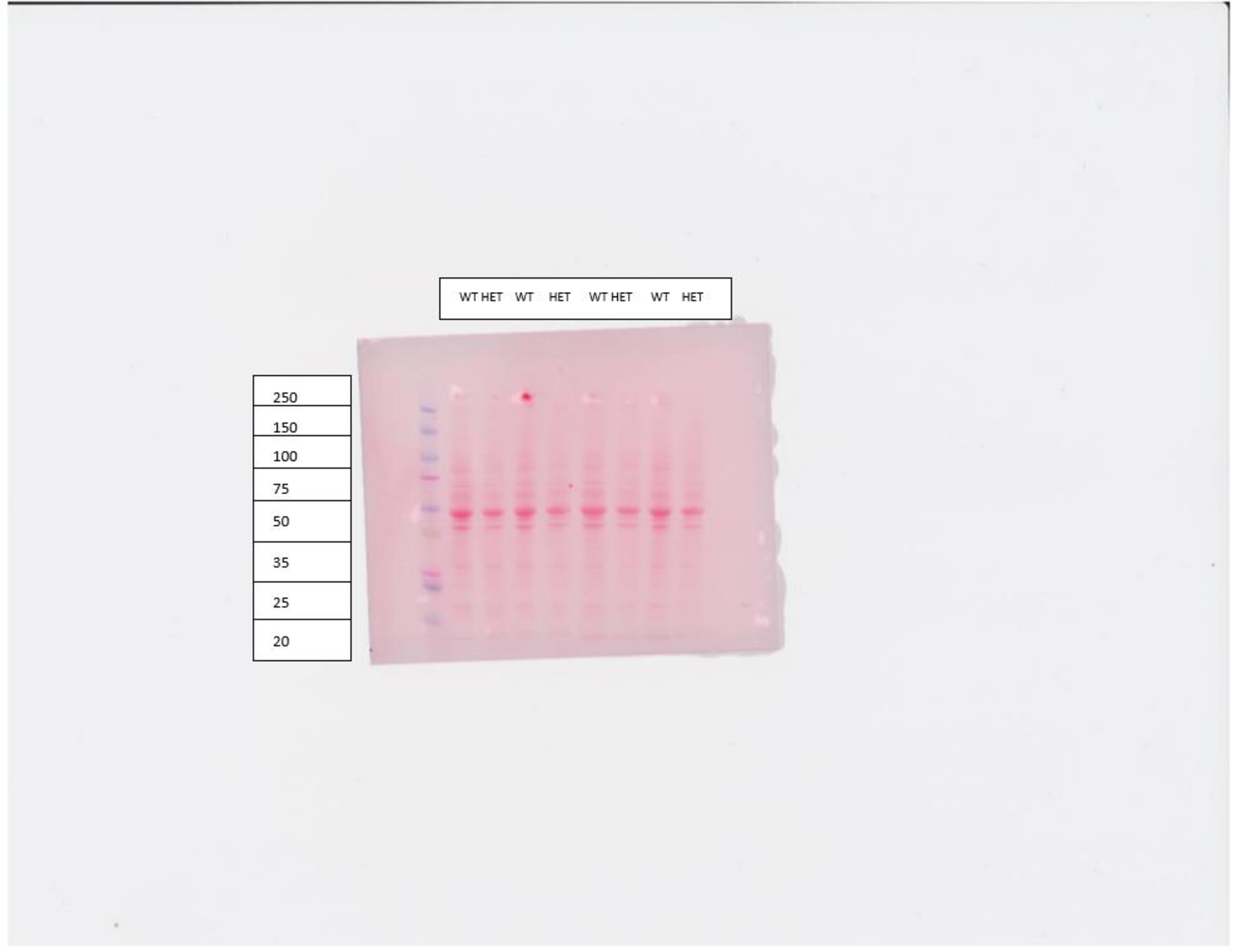

Supplement: S1 Raw image — (TIF) [file pone.0260005.s008.tif]
