# Supplementary material for: FMRP and MOV10 regulate Dicer1 expression and dendrite development
Source: PLoS One. 2021 Nov 30;16(11):e0260005. doi: 10.1371/journal.pone.0260005 (PMC8631628; doi:10.1371/journal.pone.0260005)
Supplement: Attachment — Submitted filename: Response to reviewers.docx [file pone.0260005.s009.docx]

October 28^th^, 2021

Dear Dr. Bardoni,

Thank you for this opportunity to make corrections on the manuscript. We appreciate the opportunity to respond to the comments below. Our answers are in red.

Reviewer #2: The authors greatly improved the manuscript, detailing some experimental procedures, adding complementary controls and reinforcing the discussion section. However, it still remains few points that need to be clarified:

1- Reduced expression of Mov10 leads to smaller soma size
New figure 2, supplementary figure 2 and Supplementary table 1-2
There is a discrepancy between the individual measurements of soma size area provided in the Supplementary table 1-2 and the figures.
Figure 2: Some extreme values do not appear on the scatter plot. Is it due to an error in the graph scaling or were the values discarded as outliers? In this latter case, the mean and the sem should be revised.
According to the scatter plot, the values in the WT condition in Figure 2 and Supplementary Figure 2 are different (different mean and/or sem). However, the Supplementary table 1-2 presents a single serie of WT values.
Could the statistical options for the Student t-test be detailed? Is there a Welsh correction? Is the test one or two tailed?

We looked into this and it appears to be a scaling issue when the graph is generated in our graphing software, Graphpad Prism. The software is automatically optimizing the y-axis for all data points to create a more cohesive image when extreme values are detected. In the case of Figure 2, the software excluded three outlier values and for Supplementary Figure 2 it excluded two outlier values. Each soma measurement was performed from the neurons imaged and analyzed for dendrite morphology in Figure 1, therefore only a single series of values for each genotype analyzed. We included an early version of Supplementary Figure 2 which was used for our power analysis prior to measuring all the values. We regret not catching this sooner. We have updated Figure 2B and Supplementary Figure 2. For soma size measurements, we performed a one-tailed t-test with Welch's correction. We have now specified this information clearly for all figures where that test was performed.

2- Global reduction of AGO2-associated miRNAs in the absence of FMRP
Thank you to the authors for these further details. However, I still have a question:
Lane 358-359: Using this method, we found that the significantly enriched peaks fell within 279 miRNAs (p < .001, Fig 4A, Supplementary Table 1-1).
According to the supplementary table "Ago2 eCLIP-miRNA Table" and statistical analysis provided for IP1 WT , 531 hits present an IP1 vs Input1 -log10(P-value) ≥ 3, including 523 hits with an IP1 vs. Input1 log2 Fold Change ≥ 3. How were the 279 miRNAs mentioned in the text selected? Could they be highlighted in the supplementary table?

We thank the reviewer for this question. Briefly, we de-duplicated the genes in Supplementary Table 1-1 and meant to report the number of unique miRNAs. To get this value, we selected all of the hits with -log(P-value)>3 and deduplicated the gene name list. “279” was a mistake—we meant to write “261”. The names of the 261 significantly enriched miRNAs can be found on the second sheet of Supplementary Table 1-1 named “Sig. enriched miRNAs”. We corrected 279 enriched peaks to 261

3- Dicer1 3’UTR regulation by MOV10 and FMRP
Response to the reviewer: Finally, we normalized to the WT expression of the Dicer luciferase vector which was set to 1. We have now added a clear description to the Methods.
Thank you to the authors for these further details. Regarding these precisions, I still have a question: Figure 6 C, D, E and Supplementary Figure S5 A: Relative Luciferase Activity: If the values were normalized to the WT values, how a sem can be calculated for the WT conditions?

Thank you for pointing out our mistake: we should not have had an error bar denoting the sem for the WT value. The error bar corresponded to the pre normalization value and was incorrectly copied when the graph was remade. We have now corrected this on all relevant figures.
4- Additional point:
Student's t test is not recommended as a pots-hoc test after a significant one way anova test.

We have removed the incorrect information from the manuscript.

Reviewer #3: The authors have answered to all my concerns.
There are still a few typos.
1. Pag 14 Correct Fig 1E
2. Page 19 erase ?

We have addressed these.

Sincerely,

Stephanie Ceman, on behalf of the authors
