# Supplementary material for: FMRP and MOV10 regulate Dicer1 expression and dendrite development
Source: PLoS One. 2021 Nov 30;16(11):e0260005. doi: 10.1371/journal.pone.0260005 (PMC8631628; doi:10.1371/journal.pone.0260005)
Supplement: Attachment — Submitted filename: Response to Reviewers.docx [file pone.0260005.s010.docx]

Response to Reviewers

Thank you for this opportunity to fix mistakes and make this a stronger manuscript.

Reviewer #1:

My biggest concern is the lack of consistency across different experiments that make it hard to draw some of the conclusions. For example, the authors suggest that Dicer expression is only reduced in FMR1 KO brain but not MOV10 Het KO, but the brains they used were P2 while the spine defects of the MOV10 KO mice in Fig. 2 were observed at P14 (which match the 14 DIV neurons in Fig. 1).

1.Is it possible that MOV10 deficiency in the Het KO reduces Dicer expression at P14, which can potentially explain the spine and dendrite phenotypes?

To address this question, we performed Dicer western blots on P14 whole brains from WT and Mov10 Het and found no difference (see Supplementary Fig 3B). In addition, we performed Dicer immunostain on DIV14 cultured hippocampal neurons from WT and Mov10 Het and found no difference in overall intensity in these neurons. Thus, we hypothesize that DICER expression is controlled locally in the dendrite and spine. Unfortunately, more precise measurements of DICER localization in dendrites and spines are beyond the scope of this study.

2.Likewise, Fig. 1 examines dendrite defects in cultured hippocampal neurons while Fig. 2 examines spine defects in cortical neuron in vivo using Golgi stain. It would be much better if they correlate dendrite and spine defects in the same group of neurons (either hippocampal or cortical, in vitro or in vivo).

To expand our study of the cultured hippocampal neurons in Figure 1, we examined spines in the somatosensory cortex by Golgi staining. To address the reviewer’s concern, we re-examined the Golgi-stained hippocampal regions in those brain sections but unfortunately, it was over-saturated.

3. Furthermore, the authors claim that the spine defects in MOV10 KO are different from FMRP KO, but the spines from MOV10 KO are at P14 while the spine defects in FMR1 KO mice are implicated from studies by other labs done at different ages (4- wk old and adult, p.14). Without side-by-side comparison in the same experiments, same age and same group of neurons it Is hard to draw this conclusion.

We appreciated the reviewer’s suggestion and analyzed the brains from P14 *Fmr1* ko mice and found that they were similar to WT and that both were distinctly different from the *Mov10* Het. We now include the measurements from the age-matched *Fmr1* ko mice in Figure 3C.

4.In Fig. 6C the MOV10 KO are N2A cells and FMR1 KD is done in 293T cells. They should not be put together in the same graph for comparison.

Thank you for pointing this out: we have now made 2 figures in Figure 6 (C and D).

Other comments:

5.Fig. 1: how many independent experiments have been performed?

Neuron culture was performed on at least 3 litters from each genotype and the N was compiled from the three experiments. We have now added this information to the figure 1 legend and in the methods.

6.Fig. 2C: is there statistical significance between genotypes for mature spines and immature spines?

We performed ANOVA analysis on WT mature vs. Mov10 Het mature and did not find a significant difference between these genotypes.

7.Fig. 4B: does MOV10 KO brain show changes in the amount of Ago2-associated miRNAs compared to WT? And what do the two lines in the plot refer to?

Unfortunately, we have not evaluated the MOV10 ko brain animal because we are still developing that animal. The solid black line is the best fit of the line for a perfect correlation and the dashed blue line is the actual fit of the data. We have added this information to the Figure 4 legend.

8.Fig. 6D upper panel: Does the green signal represent MYC-Dicer OE cells? It should be stated clearly in the figure. The authors should also show the images for all three experimental conditions that match the quantification in the lower panel.

Yes, the green signal represents immunoreactivity to MYC epitope, which we have now added to the figure. We have created a new Figure 7 showing all of the experimental conditions with Dicer over-expression in both Neuro2A and in neurons.

9.Fig. 6D: Top left image of the upper panel indicates WT + Dicer OE but there is no such condition in the histogram of the lower panel. Does the blue bar represent WT + Dicer OE?

We have now included WT + Dicer OE in the new Figure 7A

10.Fig. 6E: why the bars are broken?

We were trying to plot all of the data on one graph. In response to the reviewer, we replotted the data on the same scale for the Y axis (Figure 6E).

11.Fig. 6F: representative images of the MOV10 KO and FMR1 KO neurons with or without Dicer OE should be shown.

We have now added representative images in Figure 7B and C.

12.Fig. 6F: if Dicer OE can reverse the dendrite phenotypes of MOV10 Het hippocampal neurons, the authors need to examine whether Dicer expression is indeed reduced in the Het neurons compared to WT.

We examined DICER expression in WT and MOV10 Het brain at P14 (Supplemental Fig 3B) and performed DICER immunostain on DIV14 cultured hippocampal neurons from WT and Mov10 Het and found no difference in intensity in these neurons (data not shown). Thus, we hypothesize that DICER expression is controlled locally in the dendrite and spine, which is not visualizable by any of these methods.

13.Does OE Dicer restore the reduction of soma size in MOV10 Het neurons?

We did this reanalysis but did not obtain a large enough sample size of transfected neurons to reject the null hypothesis.

14.P.3, second paragraph: the sentence is too long and should be divided into separate sentences.

We have done this.

15.I think dendrite width is seldom measured. The authors should discuss the significance of a change in dendrite width observed in their study.

This is an interesting question. We did this experiment because of the effect of MOV10 loss inNeuro2A on neurite width (Supplemental fig 1). The width reflects the maturation process as we now add the following description on the top of pg.17:

“Recent work shows that dendritic widths may be shaped by intracellular transport and forces from the cytoskeleton and the area proportionality accords with a requirement for microtubules to transport materials and nutrients for dendrite tip growth (Liao et al., 2021). Thus, reduced MOV10 levels likely perturb dendrite formation because MOV10 binds cytoskeletal mRNAs (Skariah et al, 2017).”

16.P.19: Kennedy et al. 2020 is not found in the reference list

We have fixed this.

17.P.23: “suggest a mechanism for regulating local DICER expression when MOV10 and FMRP are present”. I don’t think the authors have provided evidence showing a local dendritic (but not global) reduction of DICER.

The reviewer is correct. This is our proposed model.

18.P.23: “there is also a large number of RNAs that are unique to FMRP and MOV10 and it is likely that misregulation of these mRNAs cause the unique spine features.” As mentioned in previous comment, it is not appropriate to conclude “unique spine features” from the available data because the findings on MOV10 and FMR1 KO were not done side-by-side and the ages or even populations of neurons examined are different.

We have now done this and provided the analysis in Figure 3C.

Reviewer #2:

Major points:

1.Figure 1 A: images and in particular the morphology of the selected Mov10 Het neuron do not reflect the quantitative characterization presented in B. At least 3 images of representative neurons from each condition should be presented.

We have added these representative images to Figure 1.

2.Figure1B-E: Statistical tests should be detailed for each experiments. In particular, the number of different neuronal cultures should be specified.

Fig. 1: how many independent experiments have been performed?

We prepared neurons from 3 independent litters of each genotype and thus, 3 independent cultures. The total number, N, was compiled from the three biological experiments. We have now added this information to the Figure 1 legend and in the methods.

3.Figure 3B and Supplemental Fig 2: the number of different neuronal cultures used for statistical analysis should be specified. Could the individual measurements of soma size area be provided?

We used the neuron cultures described in Figure 1 for soma size analysis and have now included this information as Supplemental table 2.

Major points:

4.Figure 4 A: It seems that there are inconsistencies between the legend of the figure and the text. Is it GO enrichment for FMRP bound mRNA (legend) or differentially Ago2-associated mRNA in WT vs Fmr1-KO (text)? Were they identified by iCLIP or eCLIP? Where is the list available? The text refers to Kenny et al., 2020 and the references section mentions Kenny et al., 2019.

Thank you for pointing these issues out. The GO enrichment is for the 29 shared brain MOV10- and FMRP- and AGO-CLIP targets, as we now describe in the Figure 4 legend and in the Results. The MOV10 iCLIP list was published in Skariah et al 2017, the AGO2 eCLIP list was published in Kenny et al 2020 (not 2019, which we have now corrected) and the FMRP iCLIP list was published by Darnell 2011.

5.Figure 4 B/ Sup table 1: legends are not accurate: what are full line, dashed line?

We now describe the full and dashed lines in the Figure 4 legend.

The Supplementary Table 1.1 is of the significantly changed AGO2-bound miRNAs.

6.How statistical analysis were performed?

They were performed by ECLIPSE: They set a significance threshold of -log10(P-value) ≥ 3 and a log2 fold change ≥ 3, which is now added to the eCLIP methods.

7.To rule out any indirect effect, the level of the Ago2 protein in WT vs Fmr1-KO P0 brains should be checked.

We agree: the AGO2 levels are the same in Fmr1 knockout and WT brains. This was published in Supplemental Figure S6 of Kenny 2020. We now state this on the bottom of page 18.

8.The Dicer mRNA being a target of FMRP, MOV10 and Ago2, the authors analyzed the expression of the DICER protein and showed that DICER protein expression is decreased in Mov10-KO N2A cells or P2 Fmr1-KO brain extracts compare to WT (Figure 5A-B).

Major points:

To fully support the hypothesis that FMRP and MOV10 modulate the Ago2-miRNA mediated regulation of the Dicer mRNA, additional experiments are required:

What are the levels of Dicer mRNA in these different models?

Dicer 1 mRNA levels are likely to be unchanged because *Dicer1* mRNA is not among the mRNAs changed in the brains of *Fmr1* ko mice compared to WT (Korb et al., 2017; Darnell et al 2011) nor in the MOV10 knockout Neuro2A cell line (Skariah et al., 2017). We believe regulation is at the level of translation. We address this on pg. 20, line 399

9.What is the impact of the absence of FMRP on the association of the Dicer mRNA with Mov10? and conversely?

Based on our earlier work showing that FMRP facilitates loading of mRNAs onto MOV10 (Kenny et al, 2014), we would predict that in the absence of FMRP, MOV10 binds less Dicer mRNA. We look forward to exploring this question when we have the *Mov10* ko mouse in-hand.

10.What is the impact of the absence of FMRP or Mov10 on the association of the Dicer mRNA with Ago2?

We examined AGO-association with Dicer1 in the absence of FMRP in the Kenny 2020 study and found that it was reduced in the absence of FMRP but not significantly.

11.What is the purpose of Figure 5C here?

We have moved this figure to the supplementary data. It is S3.

12.As MOV10 and FMRP bind murine Dicer mRNA and human DICER mRNA respectively in the 3’UTR, the authors used a DICER1 3’UTR luciferase construct to dissect the potential role of FMRP and MOV10 in the modulation of DICER1 via the 3’UTR. They showed a decreased luciferase activity in the absence of FMRP or MOV10 proteins (Figure 6C).

Major points:

13.Figure 6: Both legend and Material and Methods sections poorly explain the luciferase assay and in particular how long and short constructs participate in the calculation of the Relative Luciferase Actvity (Could the authors explain: “Mov10 KO and Fmr1 KD samples were normalized to WT Dicer long 3’UTR and Dicer short 3’UTR values were subtracted for final graph”?)

We apologize for not clearly stating our methodology: Dicer short 3’UTR contains the luciferase reporter only and allows us to examine the effect of FMRP or MOV10 loss on expression of the luciferase coding sequence itself. This is relevant as others have reported that FMRP binds luciferase coding sequence and affects expression (Chen et al. Mol Cell 2014). Dicer long 3’UTR allows us to determine the effects of the absence of MOV10 and FMRP on the entire 3’UTR. The final results had the background expression of Dicer short 3’UTR luciferase vector subtracted from the long results to account for effects on the reporter itself. Finally, we normalized to the WT expression of the Dicer luciferase vector which was set to 1. We have now added a clear description to the Methods.

14. Many steps in mRNA biogenesis could affect the reporter protein expression. qPCR to evaluate the expression of the reporter mRNA in the different conditions would strengthen the authors hypothesis.

Thank you for this suggestion. Our goal here was to report the effect on reporter expression in the absence of both MOV10 and FMRP. We do think the reduction is at the level of RNA transcription or degradation.

15.By CLIP-seq data comparison and miRNA recognition elements (MRE) analysis, the authors determined potential MSE the in close proximity of FMRP and MOV10 binding sites in the DICER 3’UTR (Sup Figure 5).

16. Minor points:

Are mouse and human Dicer mRNA 3’UTR (FMRP binding sites, Mov10 binding sites, predicted MSE ) conserved?

Thank you for asking. Yes! They are conserved and we have included this information in Supplemental figure 5C

17.Sup Figure 5: B and C should be harmonized to facilitate the reading.

We modified Supplemental Figure 5 B and C

18.Major points:

1. Figure 6 D: Incomplete legend

We have added information about how the analysis was performed.

2. Figure 6 E: Inconsistencies between text and legend.

We have revised the figure legend to reflect the constructs introduced.

3. Figure 6 F left:

Are Mov10 Het cells cultured hippocampal neurons? 7 DIV? 14 DIV?

Yes, they are DIV7 in Figure 7

19.Were analyzed neurons detected for MYC-DICER expression? What is the level of over-expression in these neurons?

Yes. We do not know the level of over-expression because we did not have enough material to immunoblot for DICER

20.Is heterologous expression homogenous?

We looked by immunostain but it was difficult to tell. It is probably heterogeneous because it is a transfection experiment

21.How does the over-expression of DICER impact dendritic arborization in WT neurons?

We did not examine WT neurons because we did not observe a difference when Dicer was over-expressed in Neuro2A (Fig 7A)

22.Can the authors comment the difference in the numbers of intersections between WT (Figure 1), Mov10 Het over- expressing negative CT and Mov10 Het over-expressing DICER (Figure 6F)?

We believe that the differences are due to a differential experimental treatment and visualization. For example, Figure 1 was Map2 staining to maximize visualization of dendrites of DIV 14 cultures while Figure 7 was DIV 7 cultures along with exposure to Lipofectamine and plasmid DNA followed by immunostaining

23.Considering the cooperative model proposed by the authors, what is the effects of FMRP over-expression on the arborization of Mov10 Het neurons?

That is in interesting question. We have not tested FMRP over-expression; however, despite working together to regulate a subset of mRNAs, MOV10 and FMRP each regulate a large number of independent mRNAs.

24.Figure 6F right:

- Same questions as above regarding the control of MYC-DICER over-expression in analyzed neurons.Why is the negative control for FMR1-KO neurons different from the one used for Mov10 Het neurons?

The negative controls were both empty expression vectors except for the tags.

25.Can the authors comment the difference in the numbers of intersections between WT (Figure 1), Fmr1-KO over-expressing negative CT and Fmr1-KO t over-expressing DICER (Figure 6F)?

Similar to what we wrote before, we believe that the differences are due to different experimental treatments, culture times and visualization. For example, Figure 1 was Map2 staining to maximize visualization of dendrites of DIV 14 cultures while Figure 7 was DIV 7 cultures along with exposure to Lipofectamine and plasmid DNA followed by immunostain.

26. To conclude, the authors discuss the potential role of FMRP in facilitating the loading of miRNA on AGO2 (Figure 7A). In parallel, they propose a model in which MOV10 alone unwinds secondary structures containing MREs and thus facilitates AGO2 binding whereas in association with FMRP, the complex is stabilized and AGO2 association is blocked (Figure 7B). Minor point:

Could the authors discuss these antagonistic effects of FMRP on the regulation of the DICER mRNA, on one hand by facilitating the miRNA machinery assembly but on the other hand protecting DICER mRNA, in collaboration with Mov10, by inhibiting its recognition by the Ago2 complex?

Thank you for this thought-provoking question. We believe that FMRP, like most RNA binding proteins, has many different functions in the cell, based on its phosphorylation state, its binding partners and its location in the cell. We might envision that in the cell body, DICER translation is facilitated by the association of FMRP and MOV10 with its mRNA. When the DICER-AGO-MOV10-FMRP complex is transported in the dendrite to the synapse, it awaits stimulation which activates DICER to produce miRNAs locally, which associate with AGO2 through its association with FMRP. Presumably translation is occurring of FMRP-MOV10 bound mRNAs because this complex blocks AGO association. However, translation must eventually stop. Perhaps methylation of the FMRP’s RGG box releases the FMRP-MOV10 complex from the 3’UTR of synaptically localized mRNAs and AGO now associates with the mRNAs to block translation. We have now added this paragraph to the Discussion.

Reviewer #3:

This is a good paper exploring an interesting aspect related to the functional interaction between MOV10 and FMRP. There are a few concerns that need to be addressed.

1. Methods are clearly described with useful details. At page 7 the differentiation methods used for N2A cells should be described.

We added the specific details for differentiation to the Methods.

2. Abnormal morphology of dendrites in cultured hippocampal neurons from Mov10 Het and Fmr1 KO mice. The authors confirmed their previously published results (Skariah et al., 2017). They should also mention other papers addressing the same issue and obtaining similar (Braun and Segal, 2000) and different results in cultured Fmr1 KO hippocampal neurons (Jacob and Doering, 2007). Did the authors consider the contribution of glia?.

We appreciate the reviewer pointing out these references. We have now added them to the manuscript. We did consider the role of glia. We do not culture on an astrocyte feeder layer, like was often done in the past. This may be why we see defects in the *Fmr1* ko neurons. Others are now culturing without feeders and see a result similar to ours, i.e., impaired arborization of *Fmr1 ko* neurons, which we cite.

3. Figure 1. Legend of figure 1 (see also results) indicates that data are from 56, 94 and 58 neurons. However, they do not indicate how many dishes in different cultures were used to gain these results. This is important to verify how reproducible are the data in different dishes, and more importantly in different cultures.

We performed 3 independent experiments from at least 3 liters per genotype. N was compiled from the three experiments. We have now added this information to the figure 1 legend and in the methods. Neurons were cultured in 24 well plates from 3 litters from each genotype.

4. Density of dendritic spines, page 14 Why the authors examined cortical neurons in the brain instead of hippocampal neurons, considering that they want to compare in vivo and in vitro conditions? Spines could be examined in vitro as well. In addition, the region/layer of cortex examined should be indicated. They also measured the width of dendritic branches. Which part of neuron dendrite was used to measure these different parameters, width of dendrites and density and morphology of spines?

To expand our study, we examined spines in the somatosensory cortex *in vivo,* which we have now indicated in the methods, while examining dendrite morphology in hippocampal culture *in vitro*, as others have done (Galvez and Greenough, 2005). To address the reviewer’s concern, we re-examined the Golgi-stained hippocampal regions but unfortunately, it was over-saturated.

With regard to the second question, for the width measurements, it was the width of the primary branches and for the density and morphology of spines it included a 10 micron region from each level of dendrite. We have also added this to the methods

5. Reduced soma size of Mov10 Het cultured hippocampal neurons. I suggest moving this part before the ex-vivo analysis of dendritic spines, to complete the in vitro examination of hippocampal morphology first.

Thank you for this suggestion. We have done this.

6. Other papers have addressed the question whether miRNAs are differentially expressed in the Fmr1KO mice. They should be cited and discussed considering that the authors found no change in the whole brain at PO. Example:

Zhang M, Li X, Xiao D, Lu T, Qin B, Zheng Z, Zhang Y, Liu Y, Yan T, Han X. Identification of differentially expressed microRNAs and their target genes in the hippocampal tissues of Fmr1 knockout mice. Am J Transl Res. 2020 Mar 15;12(3):813-824.

PMID: 32269714; PMCID: PMC7137065.Liu T, Wan RP, Tang LJ, Liu SJ, Li HJ, Zhao QH, Liao WP, Sun XF, Yi YH, Long YS. A MicroRNA Profile in Fmr1 Knockout Mice Reveals MicroRNA Expression Alterations with Possible Roles in Fragile X Syndrome. Mol Neurobiol. 2015;51(3):1053-63. doi: 10.1007/s12035-014-8770-1. Epub 2014 Jun 7. PMID: 24906954.

Thank you for this suggestion. We have added this information to our manuscript.

7. References are not always properly cited (ex. Bolduc et al., 2008 does not refer to abnormal dendritic spines in Drosophila, but excessive protein synthesis; similarly, Kelleher and Bear, 2008 and Liu-Yesucevitz refer to mRNA translation rather than spine/dendritic dysmorphogenesis). Similarly, Batish et al., 2012 is a study addressing the mRNA travel along dendrites as single particle rather than complexes of RBPs and Contractor et al., 2015 does not address the dendritic maturation and neurite extension but the altered neuronal excitability in FXS. Sure.

We apologize for these errors and appreciate the opportunity to fix them.

8. The authors suggest that DICER reduction in Fmr1 KO and Mov10 Het may affect miRNAs locally instead than leading to a global reduction of miRNA production. The authors should discuss the opposite effect on dendritic spines in Mov10 and Fmr1 KO.

Thinking about the reviewer’s question, it would seem like FMRP and MOV10 are working together in dendrite formation in cultured neurons but have discrete functions at P14 spines. We would hypothesize that the mRNAs that regulate dendritic arborization are distinct in time and perhaps place in the brain than those regulating spine morphology.

9.Darnell 2011 is cited twice.

Thank you for pointing this out. We have removed this.
